# Cardiac magnetic resonance imaging versus transesophageal echocardiography for the evaluation of mitral valve pathology prior to surgical intervention (The MagnaSound study)

**DOI:** 10.1186/1532-429X-11-S1-P122

**Published:** 2009-01-28

**Authors:** Jennifer D Cohen, Heather S Costa, Robert J Russo

**Affiliations:** Scripps Clinic, La Jolla, CA USA

**Keywords:** Mitral Valve, Cardiac Magnetic Resonance, Mitral Valve Repair, Orifice Area, Regurgitant Fraction

## Background

Transesophageal echocardiography (TEE) is the imaging modality of choice for the assessment of mitral valve (MV) pathology prior to valve repair or replacement surgery. However, TEE is a semi-invasive procedure requiring conscious sedation and may be limited by esophageal pathology and patient discomfort. Cardiac magnetic resonance (CMR) is a noninvasive imaging technique with excellent spatial resolution and is an attractive alternative for those unable or unwilling to undergo TEE. The purpose of the present MagnaSound pilot study is to assess the feasibility of a mitral valve specific CMR protocol to measure the parameters necessary for determining the appropriateness of valve repair or replacement surgery using TEE as a reference standard.

## Methods

**Figure 1:**
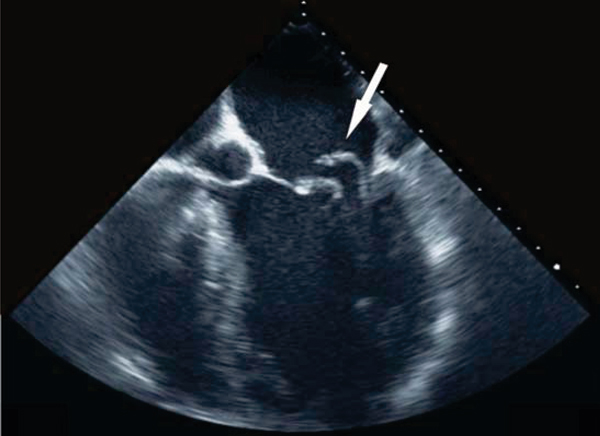
**Transesophageal echocardiogram visualizing a flail posterior leaflet of the mitral valve**.

**Figure 2:**
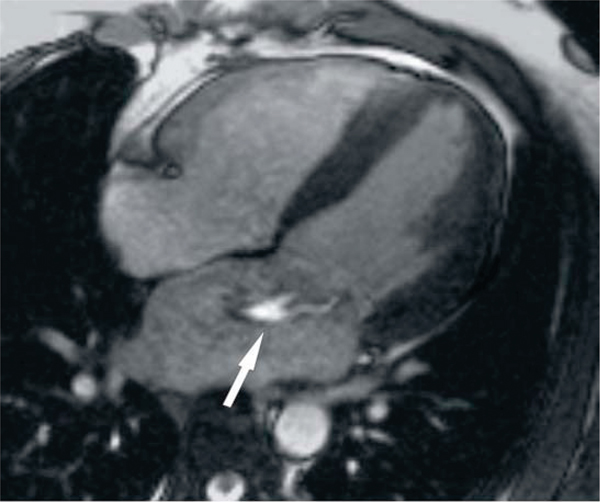
**Breath-hold cine steady-state free precession imaging in a four-chamber view visualizing the mitral regurgitation jet (flow void) due to flail a posterior leaflet**.

We performed CMR in 7 patients (57 ± 18 years) with sinus rhythm and severe MR who had undergone a clinically indicated TEE (Figure 1). CMR exams were performed within 11 days of the TEE. Using a 1.5 T Siemens Magnetom Symphony, balanced steady-state free precession (SSFP) cine images were obtained in the standard views. Additional views visualizing the papillary muscles and obtaining the maximum visualization of the regurgitant jet were performed (Figure 2). Evaluation of the maximum regurgitant jet was confirmed by gradient echo sequences with saturation bands placed across the left ventricle. Finally, phase contrast imaging sequences with velocity encoding demonstrated the mitral valve inflow patterns. Qualitative and quantitative image analysis was performed using QMass 7.0 (Medis, Inc.) and Argus (Siemens) software. The following measurements were made: regurgitant fraction, volume, and orifice area; the presence of prolapse versus flail leaflets; the length and thickness of MV leaflets; the MV inflow pattern; the number, direction and location of regurgitant jets; and three-dimensional ejection fraction, and chamber dimensions.

## Results

Data was successfully obtained in all 7 patients. Based on the results of the TEE, all 7 patients were deemed appropriate candidates for mitral valve repair surgery by the evaluating cardiac surgeon. The CMR images were interpreted by 2 physicians blinded to the TEE results. CMR correctly identified the involved leaflet and degree of impairment (prolapse versus flail) in all 7 patients compared with the TEE findings. Six of the 7 patients had a single leaflet involved. One patient had a flail posterior leaflet and a prolapsing anterior leaflet. The average maximum distance of the regurgitant jets was 4.8 ± 1.3 cm. The regurgitant fraction was 77 ± 13% and the regurgitant orifice was 0.67 ± 0.25 cm^2^ as measured by CMR. Three-dimensional ejection fraction and left ventricular dimensions were available for 5 of the 7 studies (ejection fraction 63.6 ± 9%; left ventricular end-diastolic dimension 59.9 ± 4.2 cm). CMR measurements (primary regurgitant jet, jet location and direction; leaflet lengths and thickness; MV inflow patterns; regurgitant fraction, volume and orifice area; and ejection fraction and LV dimensions) were used to assess the appropriateness of MV repair versus replacement. All 7 patients were deemed candidates for MV repair by CMR, independent of their TEE results. Both CMR and TEE correctly identified the leaflet and degree of involvement and the ability to repair the valve in the 3 patients who have since undergone surgery.

## Conclusion

This pilot study confirms that a mitral valve specific CMR protocol can adequately visualize the severity of MR and provide morphometric measurements necessary to determine a patient's candidacy for mitral valve repair versus replacement surgery. These results validate an approach to be adopted in the prospective MagnaSound study comparing TEE and CMR for the preoperative evaluation of mitral valve morphology prior to repair or replacement surgery.

